# Health-related quality of life in outpatients with chronic liver disease: a cross-sectional study

**DOI:** 10.1186/s12876-021-01890-7

**Published:** 2021-08-07

**Authors:** Domenica Gazineo, Lea Godino, Virna Bui, Latifa El Mouttaqi, Eugenia Franciosi, Alessandra Natalino, Grazia Ceci, Elisa Ambrosi

**Affiliations:** 1 Azienda Ospedaliero-Universitaria S. Orsola-Malpighi, Via Albertoni 15, 40138 Bologna, Italy; 2 Azienda USL, Via Castiglione 29, 40124 Bologna, Italy; 3 Department of Diagnostics and Public Health, University of Verona, Strada Le Grazie 8, Istituti Biologici Blocco B, 37134 Verona, Italy

**Keywords:** Chronic liver disease, Health-related quality of life, Depressive symptoms, Surveys and Questionnaires, Cross-sectional studies

## Abstract

**Background:**

The symptoms and complications related to chronic liver disease (CLD) have been shown to affect patient well-being. Currently there is limited research data on how CLD severity may affect both health-related quality of life (HRQOL) and the development of depressive symptoms in CLD patients. Moreover, the ongoing advances in CLD treatment, and its effect on HRQOL, highlight the need for further studies. Therefore, the aim of the present study was to evaluate if the CLD severity may affect the HRQOL and the development of depressive symptoms.

**Methods:**

A cross-sectional study was conducted. Patients with CLDs were identified at their regular visits to the outpatient clinic of the Sant’Orsola-Malpighi Hospital in Bologna, between September 2016 and July 2017. HRQOL was measured with Short Form 12 (SF-12) and Nottingham Health Profile (NHP) questionnaires; depressive symptoms were measured with Beck Depression Inventory-II (BDI). CLD severity was measured using the MELD score and the sample was stratified into five classes according to it. Group comparisons were conducted using the Kruskal–Wallis test.

**Results:**

Two hundred and fifty-four patients were included. Mean age was 62.84 years (SD 11.75) and 57.9% were male. Most participants were affected by compensated cirrhosis (140.2%) and chronic hepatitis (40.2%), with a disease duration ≥ 5 years (69.3%). Regarding the MELD score, 67.7% of patients belonged to Class I, 29.9% to Class II, and 2.4% to Class III. There were not patients belonging to the Classes IV and V.

No statistically significant differences were found in all SF-12 and NHP domains between the MELD classes, except for CLD impact on sexual life and holidays (*p* = 0.037 and *p* = 0.032, respectively). A prevalence rate of 26% of depressive symptoms was reported, no statistically significant differences were found in BDI-II total scores between the three MELD classes.

**Conclusions:**

All domains of HRQOL and depression were altered in CLDs patients, nevertheless CLD severity was not confirmed as an affecting factor for HRQOL.

## Background

Chronic liver diseases (CLDs) are considered a major public health burden at a global level [1, 2]. A significant increase in morbidity and mortality from CLDs has been observed worldwide over the last decades in contrast to a decrease in cardiovascular diseases, which instead were constituting the main critical health challenge in many industrialized countries during the second half of the twentieth century [3–6]. Available data suggest that 29 million people in Europe are currently affected by CLDs, with an estimated burden of 170,000 deaths per year attributed to CLDs [7]. The main causes of cirrhosis and liver cancer in Europe are viral hepatitis B and C, excessive alcohol consumption and metabolic syndrome [8, 9]. The disease manifestations related to cirrhosis and other CLDs, such as ascites, hepatic encephalopathy, recurrent variceal bleeding, fatigue, joint pain, abdominal pain, muscle cramps, skin itching, loss of appetite, depression and anxiety, have been shown to negatively affect patient well-being and health-related quality of life (HRQOL) [1, 2, 10, 11].

Moreover, CLDs are linked to job loss, impaired functioning, and low self-esteem [12–16].

The HRQOL is a broad concept which reflects the perception of patients on how the effects of disease and treatment impact on their mental well-being, physical health, functional status, social relationships, personal beliefs’ and overall [1, 2, 17].

With the recent therapeutic advances, the long-term survival in CLDs has improved; therefore, many individuals, even those who undergo liver transplantation, may live a significant proportion of their life with advanced CLD [18, 19].

Thus, HRQOL has become, beyond more traditional clinical endpoints like mortality rates, biochemistry results and incidence of complications [8, 18], an increasingly important outcome in this patient population.

Given the increased burden of CLDs, as well as the increased awareness of patient reported outcomes, a robust assessment of HRQOL and possible related variables could help healthcare professionals to provide services taking into account clinical and patient-related factors in a more balanced way, in order to better tailor CLD treatments and to identify targets for new therapies [18].

At the same time, CLDs have been long recognized and associated with depression [20] with an occurrence reported in up to 15% of patients waiting for a liver transplant and in up to 57% of patients with cirrhosis [21]. Depressive symptoms have been associated with reduced HRQOL and worsened cognitive function [21]. CLDs severity is normally considered by physicians an important prognostic factor, and previous studies found that CLDs severity has an impact on patient’s HRQOL, affecting both physical and psycho-social aspects. [12, 14]. However, to the best of our knowledge, few studies have been conducted, especially in the Italian context, on how CLDs severity influences [12, 14, 22–24] both physical and psycho-social aspects of HRQOL, such as self-care, daily life activities, and depression.

Moreover, considering the ongoing advances in CLD treatment and its effect on HRQOL, further studies looking at HRQOL and depressive symptoms in patients with CLDs, are needed.

Therefore, the aim of the present study was to evaluate if CLD severity may influence the HRQOL and lead to the development of depressive symptoms. We expect that the severity of disease may be related to a reduced perception of HRQOL and to an increased incidence of depressive symptoms.

The results of this study could be used to develop interventions and policies aiming to improve quality of life for CLD subjects.

## Methods

### Design

A cross sectional study design based on three questionnaires was employed.

### Setting

This study was carried out at the CLD Outpatient Clinic of the Sant’Orsola-Malpighi Hospital (Bologna), whose clinical and research activities are aimed at the treatment of chronic liver diseases and the prevention/treatment of their complications. The Clinic takes care of patients with advanced liver disease which are candidates for liver transplantation and post-transplant follow-up.

### Sampling and participants

Patients were identified as possible candidates for the study during their regular visits to the CLD Outpatient Clinic. All patients aged 18 years or older, with a diagnosis of CLD (compensated or decompensated cirrhosis, chronic hepatitis B, C, D, E, autoimmune hepatitis, Primary biliary cholangitis (PBC), Primary sclerosing cholangitis (PSC), or Hepatocellular Carcinoma (HCC)), consecutively admitted during the study period (September 2016–July 2017) to the Clinic, able to understand and communicate in Italian language, and willing to participate, were included.

Those patients with alcoholic liver disease and with overt encephalopathy (grade II or more), as according to the West-Haven criteria for grading mental state [25], were excluded.

### Measurements and data collection

The following variables were collected by the RN at inclusion:

- demographic data, such as age, gender, education level and marital status;
- patient’s clinical history: CLD duration (< 5 years or ≥ 5 years), presence of comorbidities as measured with the Charlson Index [26], number, motivation and length of stay of hospital admissions during the last 12 months, previous variceal bleeding, presence of portacaval shunt or TIPS;
- CLD signs and symptoms: presence and severity of clinically detectable ascites, of clinically detectable encephalopathy, as measured with West-Haven criteria [21], presence of general malaise, anorexia, weakness and fatigue, low grade fever, jaundice, splenomegaly, fluid retention, arthralgia, pruritus and muscle cramps over the last month;
- severity of CLD, as measured with MELD score using the original mathematical formula: 9.57 × loge (creatinine) + 3.78 × Loge (total bilirubin) + 11.2 × Loge (INR) + 6.43 [27]. Patients were categorized into five groups, as proposed by Wiesner and colleagues [28], assuming that an higher MELD score would indicate a worse degree of liver disease: class I (≤ 9), class II (10–19), class III (20–29), class IV (30–39), class V (≥ 40).

Aiming at evaluating HRQOL a questionnaire was administered to all consented 254 patients at the end of the visit at Outpatient Clinic of CLD. The questionnaire consisted of the following three scales: (1) the Short Form 12 Questionnaire (SF-12), (2) the Nottingham Health Profile (NHP), (3) and the Beck Depression Inventory- II (BDP-II), in their Italian validated versions [29–31].

The SF-12 [29] is a 12-item health survey, developed from the original SF-36 [8]. It covers four domains in the area of physical health, including physical functioning (e.g. limitations in daily life due to health problems), role-physical (e.g. role limitations due to physical health problems), bodily pain (e.g. pain frequency and interference with usual roles) and general health (e.g. individual perceptions of general health), and four in the mental health area, including vitality (e.g. energy levels and fatigue), social functioning (e.g. limitations of social activities due to health interferences), role-emotional (e.g. role limitations due to emotional problems), and mental health (e.g. psychological distress). It produces two summary measures, a Physical Component Summary (PCS-12) and a Mental Component Summary (MCS-12), calculated by summing factor-weighted scores across all eight sub-scales. PCS and MCS range from 0 (lowest level of perceived health) to 100 (highest level of perceived health).

A score of 50 or more indicates a positive self-rated health, while a score below 50 indicates a negative perception [29].

The NHP [30] is a generic health status questionnaire designed to measure patient’s perceived emotional, social, and physical health. It consists of two parts, the first one comprises 38 items and focuses on individual health status and includes energy levels (three items), pain (eight items), sleep (five items), mobility (eight items), emotional reaction (nine items) and social isolation (five items). The second part addresses the impact of illness on daily life and it consists of seven items that cover the seven life domains regarding occupation, housework, social life, family life, sexual function, hobbies and holidays. All items have a dichotomous answer option (yes/no) and each section score is weighted from 0 (best health state) to 100 (worst health state).

The BDP-II [31] is a scale comprising 21 self-evaluation items assessing the severity of common depressive symptoms, 13 of which cover cognitive-affective symptoms, such as sadness, pessimism, past failure, loss of pleasure, guilty feelings, punishment feelings, self-dislike, self-blame, suicidal thoughts or wishes, crying, withdrawal, indecisiveness, and physical appearance concerns; and eight cover somatic symptoms, such as irritability, work ability, sleep disturbances, tiredness or fatigue, appetite disturbances, weight fluctuations, health concerns and lack of sexual interest.

For each item, participants are required to choose the point scale (from 0 to 3) that best describes how they felt in the last 2 weeks. The total score ranges from 0 to 63, with higher scores reflecting higher levels of depression.

### Statistical analysis

Data were entered anonymously into a dedicated database. Kolmogorov–Smirnov test was carried out to evaluate the distribution of the variables. Baseline characteristics, including patient demographics data, patient’s clinical history, CLD signs and symptoms and severity of CLD measured using the MELD score, were summarized as means, standard deviations and ranges for continuous variables and percentages for categorical variables.

The Pearson’s chi-squared and the Kruskal–Wallis one-way analysis of variance were used respectively with variables with parametric and nonparametric distribution.

The Kruskal–Wallis one-way analysis of variance was performed to compare PCS-12, MCS-12 and NHP-Part I mean scores among MELD classes. A post-hoc tests using the Bonferroni correction, plotted histograms and a scatter plot were performed if significant differences were noted.

The Pearson’s chi-squared test was run to determine the correlation between MELD score and NHP-Part II.

All statistical analyses were done using SPSS computer software for Windows 19.0 (IBM Corp., Armonk, NY, USA). Differences were considered statistically significant if the *P* value was < 0.05.

## Results

### Socio-demographic and clinical characteristics

**Fig. 1:**
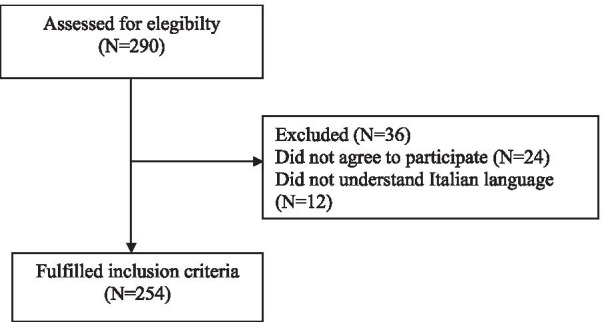
Patient flow chart

The patient flowchart is summarized in Fig. 1. The socio-demographic and clinical characteristics of the 254 CLD patients who consented to enroll in the study are shown in Table 1.

**Table 1:** Patient sample characteristics

	All patients (N = 254)	MELD score*		
	n (%)	Class I (N = 178) n (%)	Class II (N = 76) n (%)	Class III (N = 6) n (%)
Age mean (SD), range	62.84 (11.75) (33–86)	62.28 (11.58) (34–86)	63.51 (12.02) (33–86)	70.17 (12.07) (50–82)
Females	107 (42.1)	81 (47.1)	24 (31.6)	2 (33.3)
*Marital status (N* = *249)*				
Married	165 (65.0)	108 (64.3)	53 (69.7)	4 (66.7)
Divorced	34 (13.4)	21 (12.5)	12 (15.8)	1(16.7)
Widowed	29 (11.4)	20 (11.9)	8 (10.5)	1 (16.7)
Unmarried	21 (8.3)	18 (10.7)	3 (3.9)	0
*Level of education (N* = *249)*				
Primary school	49 (19.7)	31 (18.6)	17 (22.4)	1 (16.7)
Secondary school	85 (33.5)	52 (31.1)	30 (39.5)	3 (50.0)
High school	87 (34.3)	62 (37.1)	23 (30.3)	2 (33.3)
University	28 (11.0)	22 (13.2)	6 (7.9)	0
*Nationality*				
Italian	232 (91.3)	157 (91.3)	69 (90.8)	6 (100.0)
Other	22 (8.7)	15 (8.7)	7 (9.2)	0
*Type of CLD*				
Compensated cirrhosis	102 (40.2)	58 (33.7)	40 (52.6)	4 (66.7)
Chronic hepatitis B, C, D, E virus infection	102 (40.2)	79 (45.9)	22 (28.9)	1 (16.7)
Autoimmune hepatitis Primary biliary cholangitis (PBC) and Primary sclerosing cholangitis (PSC)	24 (9.4)	20 (11.6)	4 (5.3)	0
Hepatocellular Carcinoma (HCC)	14 (5.5)	10 (5.8)	4 (5.3)	0
Decompensated cirrhosis	12 (2.9)	5 (7.9)	6 (16.7)	1 (0.4)
*Duration of CLD*				
≥ 5 years	176 (69.3)	124 (72.1)	48 (63.2)	4 (66.7)
Hospital stay during the last 12 months (days), mean (SD) (N = 68)	13.44 (22.46)	13.09 (28.72)	14.15 (15.68)	7.00 (0.00)
*Comorbidity Index*				
0	115 (45.3)			
1	40 (15.7)	28 (32.9)	11 (22.4)	1 (16.7)
2	11 (4.3)	7 (8.2)	2 (4.1)	2 (33.3)
3	52 (20.5)	27 (31.8)	24 (49.0)	1 (16.7)
4	14 (5.5)	8 (9.4)	5 (10.2)	1 (16.7)
5	10 (3.9)	5 (5.9)	4 (8.2)	1 (16.7)
6	6 (2.4)	4 (4.7)	2 (4.1)	0
7	5 (2.0)	5 (5.9)	0	0
8	1 (0.4)	0	1 (2.0)	0
Previous variceal sclerosis	68 (26.8)	31 (18.0)	35 (46.1)	2 (33.3)
Portacaval shunt or TIPS	11 (4.3)	6 (3.5)	5 (6.6)	0
*Clinically detectable ascites (N* = *247)*				
Absent	192 (75.6)	146 (88.0)	43 (57.3)	3 (50.0)
Mild-to-moderate	7 (2.8)	2 (1.2)	5 (6.7)	0
Severe	8 (3.1)	3 (1.8)	5 (6.7)	0
Under loop-diuretic treatment	37 (14.6)	13 (7.8)	22 (29.3)	2 (33.3)
Under chronic albumin infusion	3 (1.2)	2 (1.2)	0	0
*Clinically detectable encephalopathy (West-Haven criteria)*^a^				
Grade I	13 (5.1)	10 (2.3)	8 (10.5)	1 (16.7)
None	241 (94.9)	168 (97.7)	68 (89.5)	5 (83.3)
*CLD signs and symptoms (last month)*				
Muscle cramps	79 (31.1)	50 (29.1)	27 (35.5)	2 (33.3)
Pruritus	60 (23.6)	31 (18.0)	27 (35.5)	2 (33.3)
Weakness and fatigue	47 (18.5)	32 (18.6)	15 (19.7)	0
Splenomegaly	46 (18.1)			
Arthralgia	42 (16.5)	29 (16.9)	11 (15.5)	2 (33.3)
General malaise	42 (16.5)	29 (16.9)	13 (17.1)	0
Fluid retention	29 (11.4)	17 (9.9)	11 (14.5)	1 (16.7)
Low grade fever	9 (3.5)	5 (2.9)	3 (3.9)	1 (16.7)
Jaundice	7 (2.8)	1 (0.6)	4 (5.3)	2 (33.3)
Anorexia	4 (1.6)	3 (1.7)	1 (1.3)	0

SD, Standard deviation; CLD, Chronic liver disease; TIPS,Transjugular intrahepatic portosystemic shunt

*MELD score using the original mathematical formula: 9.57 × loge(creatinine) + 3.78 × Loge(total bilirubin) + 11.2 × Loge(INR) + 6.43 [21]. Patients were categorized into three groups, as proposed by the Wiesner and colleagues [27], where a higher MELD score indicates a worse degree of liver disease: class I (≤ 9), class II (10–19), class III (20–29), class IV (30–39)

^a^West-Haven criteria: grade I—trivial lack of awareness/sleep disorders -, grade II—lethargy -, grade III—somnolence to stupor -, grade IV—coma

No significant correlations were observed with CLD type and mean age (χ^2^ 3.815; df = 2 *p* = 0.148) and mean hospital stay during the last 12 months (χ^2^ 1.508; df = 2 *p* = 0.470). Based on these results, we decided to stratify our population only by MELD classes.

### MELD classes

Regarding MELD score, 172 (67.7%) patients belonged to Class I, 76 (29.9%) to Class II, and six (2.4%) to Class III. No patients belonged to Class IV (30–39) and V (≥ 40).

### Short Form 12 Questionnaire

The mean score for our participants were 44.31 for the PCS-12 and 45.17 for the MCS-12. Table 2 shows the results from the comparison of PCS-12 and MCS-12 among MELD classes, and no differences were observed.

**Table 2:** Comparison of Short Form 12 Questionnaire (SF-12) among MELD score

	MELD score*			All patients (N = 254)	*P*-value^a^
	Class I (N = 178) Mean (SD)	Class II (N = 76) Mean (SD)	Class III (N = 6) Mean (SD)	Mean (SD)	
PCS-12^b^	45.13 (10.40)	42.94 (10.22)	38.49 (12.00)	44.31 (10.43)	0.080
MCS-12^c^	45.49 (11.51)	44.62 (12.25)	43.02 (14.65)	45.17 (11.78)	0.810

*MELD score using the original mathematical formula: 9.57 × loge(creatinine) + 3.78 × Loge(total bilirubin) + 11.2 × Loge(INR) + 6.43 [21]. Patients were categorized into three groups, as proposed by Wiesner and colleagues [27], where a higher MELD score indicates a worse degree of liver disease: class I (≤ 9), class II (10–19), class III (20–29)

^a^Kruskal-Wallis one-way analysis of variance by ranks

^b^Physical Component Summary scale (PCS-12)

^c^Mental Component Summary scale (MCS-12)

### Nottingham Health Profile

Among the six domains of NHP—Part I (individual health status), the highest score of 29.66 (SD 35.23) was related to energy levels, while the lowest score of 14.05 (SD 23.80) was related to social isolation. No differences were observed in the NHP—Part I mean scores between the MELD classes (Table 3).

**Table 3:** Comparison of Nottingham Health Profile – Part I among MELD score

NHP – Part I Individual health status	MELD score*			All patients (N = 254)	*P* value^a^
	Class I (N = 176) Mean (SD)	Class II (N = 76) Mean (SD)	Class III (N = 6) Mean (SD)	Mean (SD)	
Mobility	18.75 (21.81)	23.29 (23.77)	35.91 (27.11)	20.51 (22.66)	0.142
Energy levels	26.36 (33.48)	35.80 (37.97)	46.61 (39.30)	29.66 (35.23)	0.105
Pain	14.08 (24.80)	14.42 (21.96)	41.67 (47.49)	14.83 (24.92)	0.289
Sleep	19.78 (27.92)	22.70 (27.01)	26.94 (26.25)	20.82 (27.56)	0.305
Emotional reactions	19.17 (23.92)	20.11 (23.36)	16.22 (29.16)	19.38 (23.78)	0.756
Social isolation	12.89 (23.15)	16.82 (25.51)	12.04 (20.21)	14.05 (23.80)	0.328

*MELD score using the original mathematical formula: 9.57 × loge(creatinine) + 3.78 × Loge(total bilirubin) + 11.2 × Loge(INR) + 6.43 [21]. Patients were categorized into three groups, as proposed by the Wiesner and colleagues [27], where a higher MELD score indicates a worse degree of liver disease: class I (≤ 9), class II (10–19), class III (20–29)

^a^Kruskal-Wallis one-way analysis of variance by ranks

Comparing the NHP—Part II mean scores between the MELD classes, a statistically significant difference emerged from sex life (χ^2^ 6.610; df = 2, *p* = 0.037) and vacations (χ^2^ 6.914, df = 2, *p* = 0.032) (Table 4).

**Table 4:** Comparison of Nottingham Health Profile—Part II among MELD score

NHP – Part II Life areas affected	MELD score*			Total (N = 252)	P-value^a^
	Class I (N = 176) n (%)	Class II (N = 76) n (%)	Class III (N = 6) n (%)	n (%)	
Item 43—Is your present state of health causing problems with your sex life?					
*YES*	33 (18.7)	24 (31.6)	3 (50.0)	60 (23.8)	0.037
Item 45—Is your present state of health causing problems with your vacations (summer or winter vacations, weekends away, etc.)?					
*YES*	39 (22.1)	15 (19.7)	4 (66.6)	58 (23.0)	0.032

*MELD score using the original mathematical formula: 9.57 × loge(creatinine) + 3.78 × Loge(total bilirubin) + 11.2 × Loge(INR) + 6.43 [21]. Patients were categorized into three groups, as proposed by the Wiesner and colleagues [27], where a higher MELD score indicates a worse degree of liver disease: class I (≤ 9), class II (10–19), class III (20–29)

^a^Pearson’schi-squared test

### Beck Depression Inventory—II

The prevalence of depressive symptoms was of 26% (N = 66). Higher BDI-II mean scores were found for somatic symptoms (6.50 ± 5.71), while lower BDI-II mean scores were obtained for cognitive-affective symptoms (3.64 ± 4.68). No differences were observed in the BDI-II mean scores between the MELD classes in both cognitive-affective (χ^2^ 1,537, df = 2, *p* = 0.464) and somatic symptoms BDI-II domains (χ^2^ 2,203, df = 2, *p* = 0.332) (Table 5).

**Table 5:** Comparison of Beck Depression Inventory-II (BDI-II) among MELD score

BDI-II	MELD score*			All patients (N = 254)	*P*-value^a^
	Class I (N = 172) Mean (SD)	Class II (N = 76) Mean (SD)	Class III (N = 6) Mean (SD)	Mean (SD)	
Cognitive-affective symptoms	3.67 (4.58)	3.66 (4.93)	2.33 (4.76)	3.64 (4.68)	0.464
Somatic symptoms	6.10 (5.30)	7.18 (6.34)	9.33 (7.92)	6.50 (5.71)	0.332

*MELD score using the original mathematical formula: 9.57 × loge(creatinine) + 3.78 × Loge(total bilirubin) + 11.2 × Loge(INR) + 6.43 [21]. Patients were categorized into three groups, as proposed by Wiesner and colleagues [27], where a higher MELD score indicates a worse degree of liver disease: class I (≤ 9), class II (10–19), class III (20–29)

^a^Kruskal-Wallis one-way analysis of variance by ranks

Considering each item, a statistically significant difference was observed for the following symptoms: loss of pleasure (χ^2^ 12,950, df = 6, *p* = 0.044), suicidal thoughts or wishes (χ^2^ 42.130, df = 6, *p* = 0.000), agitation (χ^2^ 13.715, df = 6, *p* = 0.033), concentration (χ^2^ 17.892, df = 6, *p* = 0.007) and loss of interest in sex (χ^2^ 15.558, df = 6, *p* = 0.016).

## Discussion

Health-related quality of life is one of the most important aspects in medicine and its measurement is crucial, because it constitutes an essential measure to assess the effectiveness of medical care and the related health outcome for the patient: measuring patients’ perception and the extent to which they can actually function in their daily activities are very important when the main objective of treatment is to improve how the patient feels. The first relevant finding of the study is that our sample of Italian CLD patients revealed a greatly impaired perceived health status involving both physical and mental health, as measured with SF-12 and NHP. This is in line with the results of previous studies reporting that it is expected to find impaired quality of life in patients with CLDs [1, 2].

Concerning the comparison of all SF-12 and NHP-part 1 domains between the MELD classes, no significant differences were found. And thus, our hypothesis that disease severity influences the degree of HRQOL impairment was not confirmed. This result is in contrast with previous studies [12–14] and it might be explained by the fact that most of the included patients were classed as MELD score Class I.

Considering HRQOL assessed by NHP -part I, the main indicator of worse quality of life regarded a reduction in energy levels. This was an expected outcome, as fatigue represents one of the most frequent and disabling CLD symptoms and a confirmed independent predictor of low HRQOL [12]. On the contrary, the best HRQOL score was found in the Social Isolation domain, which could relate to sample characteristics. Almost 66% of patients were married and this might have helped them to feel less lonely and isolated.

Considering the seven items of NHP-part II, the impact of CLD on sexual life and holidays was mostly reported by patients belonging to the MELD Class I. A possible explanation for this finding could be that the patients in the MELD score Class I were younger (MELD I: 62.2 ± 11.7 years vs MELD II: 63.5 ± 12.02 and MELD III: 70.2 ± 12.1; data not shown) and thus they valued more these activities compared to older patients. The influence of CLD on sexual functioning has been previously reported [14, 31], in fact Remy and colleagues [32] demonstrated that reduction in the quality of life was frequent and was associated with psychological disorders, reduced sexuality and apprehension of the future. Marchesini and colleagues [14] also showed a significant difference in sexual life comparing subjects with cirrhosis to a random sample of Italian population (42.3 *versus* 18.7, *p* < 0.001).

Furthermore, our research highlighted that also the life domain related to vacations is affected by the disease and these results represent novel data regarding the compromised quality of life of CLD patients.

Moreover, our findings showed a prevalence rate of 26% of depressive symptoms in CLD patients, as measured with BDI-II; this was broadly inferior when compared with previous studies where the literature reported up to 57% of patients suffering from depression, ranging from mild to extremely severe depression [21, 33]. However, these data referred to specific CLD populations, such as patients with chronic hepatitis C virus infection [34] and cirrhosis [21, 33, 34], where psychological (e.g. stigmatization of having a chronic infectious disease) and biological (e.g. HCV biological role and anti-viral treatment) theories have been developed to explain their association with depression [35].

Similarly, to the results on HRQOL, it seems that no differences exist in BDI-II total scores between the three MELD classes; thus suggesting that disease severity does not influence the onset of depressive symptoms.

Considering the individual items, the main indicators of worse HRQOL were loss of pleasure, suicidal thoughts or wishes, irritability, inability to work and lack of sexual interest. BDI-II scores increased as the patients reached a higher MELD class, this phenomenon is probably due to the presence of debilitating clinical symptoms related to the severity of liver impairment [33].

Some limitations of this study must be mentioned. Considering sample characteristics, we enrolled patients solely from one outpatient clinic, we collected data from a small sample, with different types of CLD and disparity in numbers among subgroups (e.g. MELD score classes), which might have contributed to some bias in revealing differences in HRQOL. We excluded patients with overt hepatic encephalopathy, and this might have contributed to a selection bias; nevertheless, this decision is due to the fact that the cognitive impairment related to the overt hepatic encephalopathy may affect patient’s answers to the questionnaire.

We did not measure some variables which have been previously associated with HRQOL, such as type of comorbidities and treatments, while the cross-sectional nature of the study prevented a clear definition of the cause and the effect of the variables considered. Finally, we did not assess a pre-existing diagnosis of depression and use of antidepressants, which might have influenced BDI-II scores. Despite these limitations, we evaluated HRQOL of CLD through validated methods and the results of this study can be compared with other National and International data. Moreover, to the best of our knowledge, this is the first study that has explored the HRQOL of CLD using the SF-12, the NHP and the BDP-II questionnaires in the Italian context.

## Conclusion

In this study, we analysed the influence of CLD disease severity, as measured with MELD score, on HRQOL impairment and development of depressive symptoms. As a result, all domains of HRQOL were altered in CLDs and about one third of involved patients reported depressive symptoms, nevertheless disease severity was not confirmed as an affecting factor, except for the impact on sexual life and vacations. Thus, the initial hypothesis that severity of disease is related to a reduced perception of HRQOL and an increased development of depressive symptoms was not confirmed.

## Data Availability

The datasets used and/or analysed during the current study are available from the corresponding author on reasonable request.

## Abbreviations

HRQOL: Health-related quality of life
CLD: Chronic liver disease
PBC: Primary biliary cholangitis
PSC: Primary sclerosing cholangitis
HCC: Hepatocellular carcinoma
SF-12: Short Form 12 Questionnaire
NHP: Nottingham health profile
BDP-II: Beck Depression Inventory-II
PCS-12: Physical component summary
MCS-12: Mental component summary
